# Mechanisms of Peritoneal Mesothelial Cells in Peritoneal Adhesion

**DOI:** 10.3390/biom12101498

**Published:** 2022-10-17

**Authors:** Ruipeng Wang, Tiankang Guo, Junliang Li

**Affiliations:** 1 The First School of Clinical Medical, Gansu University of Chinese Medicine, Lanzhou 730030, China; 2 Department of General Surgery, Gansu Provincial Hospital, Lanzhou 730030, China; 3 The First School of Clinical Medicine, Lanzhou University, Lanzhou 730030, China

**Keywords:** peritoneal adhesions, peritoneal mesothelial cells, mesothelial–mesenchymal transition, inflammation, fibrosis

## Abstract

A peritoneal adhesion (PA) is a fibrotic tissue connecting the abdominal or visceral organs to the peritoneum. The formation of PAs can induce a variety of clinical diseases. However, there is currently no effective strategy for the prevention and treatment of PAs. Damage to peritoneal mesothelial cells (PMCs) is believed to cause PAs by promoting inflammation, fibrin deposition, and fibrosis formation. In the early stages of PA formation, PMCs undergo mesothelial–mesenchymal transition and have the ability to produce an extracellular matrix. The PMCs may transdifferentiate into myofibroblasts and accelerate the formation of PAs. Therefore, the aim of this review was to understand the mechanism of action of PMCs in PAs, and to offer a theoretical foundation for the treatment and prevention of PAs.

## 1. Introduction

A peritoneal adhesion (PA) is a pathological fibrous band between visceral organs or between organs and the abdominal wall [1,2]. Surgery, trauma, chronic peritoneal inflammation, peritoneal dialysis, endometriosis, etc., can induce PA formation [3,4,5,6,7]. However, most PAs are caused by surgery [8,9,10]. PAs can cause pain, female infertility, intestinal obstruction, and other problems, and bring difficulties to reoperation [6,11,12]. Presently, there is no effective strategy to prevent and treat PAs. A thin layer of cells called peritoneal mesothelial cells (PMCs) covers the outside of the peritoneum; these serve as the primary barrier of the abdominal cavity [13]. PMCs may have a significant role in the occurrence and progression of PAs [2,14,15,16,17]. The aim of this review is to provide a new perspective on PA prevention and treatment by summarizing the mechanism of PMCs in PA formation.

## 2. Characteristics and Functions of PMCs

### 2.1. Anatomical Features of PMCs

Among the three serous cavities in the human body, the peritoneal cavity is the largest and most complex [7,18,19]. Usually, the visceral peritoneum covers the visceral and mesenteric surfaces of the abdomen, and the parietal peritoneum covers the abdominal wall and the inner surface of the pelvis. The visceral and parietal peritoneum surround the peritoneal cavity [19]. The peritoneum is covered by a thin layer of mesothelial cells [7,20]. Below the mesothelial cells, the basement membrane and interstitial subcutaneous tissue, including collagen fibers, blood vessels, and fibroblasts, are found [21,22]. PMCs have apical–basal polarity, intercellular junction complexes, and apical microvilli [23]; these structures may be the most basic form of PMCs that maintain peritoneal integrity. The junction of two or more PMCs forms stomata [6], which lead to the submesothelial lymphatic system and play a role in fluid transport [24,25,26,27,28].

### 2.2. Pathophysiological Function of PMCs

The glycocalyx, composed of surfactants, phospholipids, and glycosaminoglycans distributed on top of the microvilli of PMCs, creates a lubricating environment for visceral organ activities [20,29,30]. PMCs have epithelial and mesenchymal features and can transform under physiological and pathological conditions [6]. The peritoneum is a naturally semipermeable membrane under physiological conditions [31,32,33], but after repeated peritoneal dialysis, PMCs undergo mesothelial–mesenchymal transformation (MMT), which can lead to peritoneal fibrosis [34,35,36,37]. In addition, PMCs can capture bacteria, chemical molecules, and other substances to play a protective barrier role [21]. They can initiate inflammatory responses by presenting antigens to immune cells. Moreover, they can secrete cytokines when pathogens invade and when tissues are damaged [18]. PMCs also exhibit fibrinolytic activity, which can dissolve fibrin and prevent the formation of PAs [38].

## 3. Key Steps of PA Formation

When the peritoneum is damaged by surgical trauma or infection, or is exposed to peritoneal dialysis fluid, PMCs may undergo shedding, necrosis, or apoptosis. They can release damage-associated molecular patterns (DAMPs) or pathogen-associated molecular patterns that attract other immune cells to aggregate and trigger inflammation resulting in coagulation reactions, which increase the local vascular permeability of the peritoneum. Fibrin is also released from blood vessels with immune cells at the injured site to cover the wound [39]. However, if the fibrin fails to dissolve in time during wound healing, fibroblasts will attach to the fibrin and produce collagen to form adhesive fibrotic tissue. Finally, PMCs cover the surface of the formed adhesive fibrotic tissue to complete pathological repair [40], leading to permanent PA formation [11]. The formation process of PAs is similar to the healing process of normal tissues; however, the production and dissolution of fibrin in the normal healing process are balanced. It is the excessive deposition of fibrin that leads to the final PA formation [11]. Finding the initiating factors of the inflammatory response and fibrin deposition is crucial, as this may be beneficial in controlling the formation of PAs at the source. To sum up, the formation of PAs may be a result of the overall effects of the inflammatory response, coagulation, fibrin deposition and extracellular matrix (ECM) generation [41]. Although immune cells such as neutrophils [42,43], macrophages [40,44,45], mast cells [46], and T lymphocytes [47,48] are also involved, damage to PMCs and the exposure of the basement membrane are necessary for these cells to promote PA formation [49]. As such, the role of PMCs in PAs appears to be essential.

## 4. The Mechanism of PMCs in Promoting PAs

### 4.1. Damage to PMCs Initiates PA Formation

**Figure 1:**
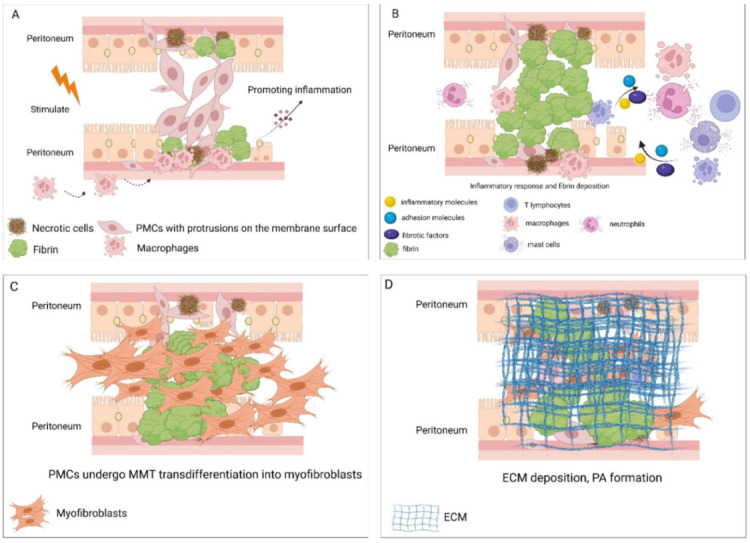
(**A**) Early stage of peritoneal adhesion (PA) formation: peritoneal mesothelial cells (PMCs) undergo shedding, necrosis, and phenotypic changes, form membrane protrusions, and fuse with each other to form early adhesions in the stimulated environment. (**B**) Intermediate stage of PA formation: PMCs initiate inflammatory responses by secreting inflammatory factors, adhesion molecules, and pro-fibrotic factors. Moreover, they are affected by the inflammatory environment, which stimulates them to further exacerbate the inflammatory process. At the same time, the fibrinolytic system is dysfunctional, causing excessive deposition of fibrin. (**C**) Late stage of PA formation: PMCs undergo mesothelial–mesenchymal transition (MMT) to form myofibroblasts. (**D**) Late stage of PA formation: myofibroblasts secrete a large amount of extracellular matrix to finally form a PA.

Smooth and intact PMCs can prevent the formation of PAs; in contrast, damaged PMCs or the shedding of PMCs may be the basis for the initiation of PAs (Figure 1A) [2,17,20]. A mouse model of PAs, induced by ligation or rubbing, suggested that PMCs with high mesothelin expression after injury may induce genes that regulate cell differentiation and proliferation, allowing PMCs to break away from the basement membrane and enter the peritoneal cavity to initiate PAs. This could be reduced by the use of anti-mesothelin antibodies [2]. After injury or activation, PMCs can remodel the ECM or directly invade the basement membrane by producing matrix metallopeptidase 2/9 (MMP-2/9) and by degrading type IV collagen [50], which may be one of the mechanisms by which PMCs enter the peritoneal cavity to induce early PA formation. Further studies have found that after PMCs are damaged, membrane protrusions and membrane fusion occur at the surface of damaged cells with the mediation of calcium ions. Concurrently, damaged PMCs release signals to adjacent normal cells, resulting in the transmission of damaged cell phenotypes and behaviors to normal cells and triggering initial PA formation [17,51]. Proliferation and scarring of PMCs subsequently occur [17]. In addition, another study has shown that fibrin is deposited at the shedding site of PMCs shortly after injury, followed by the aggregation of macrophages [39], possibly promoting inflammation (Figure 1A), which suggests that the loss of PMCs also provides an attachment point for early PAs. The above evidence indicates that the destruction of the integrity of PMCs is the initial inducement of PAs. The morphological changes and cell surface markers of PMCs in the pathological environment not only show the beginning of PA formation, but also provide attachment points for fibrin to promote PAs. Preventing the early destruction of PMCs and promoting the regeneration of PMCs in a timely manner may be an effective method to prevent the occurrence and progression of PAs.

Most PAs are caused by surgical trauma; however, other factors cannot be ignored, such as inflammation, bleeding, and peritoneal dialysis. The effect of peritoneal dialysis on PMCs may not only be mechanical damage to peritoneal catheters but also chronic stimulation of PMCs by high-sugar, acidic substances in peritoneal dialysis [52], and finally may even lead to the overall exfoliation of PMCs, aggravating the subsequent fibrotic process.

### 4.2. Dysfunction of PMCs Leads to Excessive Fibrin Deposition

To maintain the balance of the fibrinolytic system and prevent PAs under physiological conditions, PMCs can produce activating and inhibiting molecules of the fibrinolytic system, such as tissue-type plasminogen activator (t-PA), urokinase-type plasminogen activator, plasminogen activator inhibitor-1 (PAI-1), type 2 plasminogen activator inhibitor, and plasmin [1,38]. After the peritoneal membrane is damaged, a coagulation reaction begins. The aggregation of platelets at the site of vascular injury causes the cross-linking of fibrin, and with the enhancement of fibrinolytic activity, PMCs release fibrinolytic media and activate plasmin to promote fibrin dissolution, thereby promoting wound healing. After PMCs are damaged, the function of the fibrinolytic system is disrupted, and the decrease in plasminogen activation leads to insufficient production of plasmin, thereby reducing fibrinolysis and leading to PAs (Figure 1B). After surgical injury, an increase in PAI-1 levels is accompanied by a decrease in t-PA levels, and the imbalance between the two leads to fibrin deposition [53]. In addition, PAI-1 can bind to t-PA and become a chemokine by attracting macrophages to the PA site. Macrophages further enhance the secretion of PAI-1 by upregulating the receptor HER1 on PMCs, thus intensifying the deposition of fibrin at the adhesion site. The inhibition of PAI-1 promotes fibrinolysis and also prevents the recruitment of macrophages [54]. Further studies have found that adipose mesenchymal stem cell-derived extracellular vesicles, composed of a variety of proteins, DNA, mRNAs, and miRNAs [55,56,57], can alleviate PAs by promoting the healing of PMCs, making them secrete more t-PA and reducing the production of PAI-1 [58]. The above evidence suggests that the dysfunction of PMCs leads to excessive deposition of fibrin, while the recovery of the functions of PMCs helps to maintain the balance of the fibrinolytic system, thus promoting wound healing and reducing PAs.

### 4.3. PMCs Regulate the Inflammatory Process of PA Formation

PA formation is accompanied by the occurrence and development of an inflammatory reaction. PMCs induce inflammation by producing inflammatory molecules, adhesion molecules, and pro-fibrotic factors to accelerate the formation of PAs (Figure 1B) [38,42,59]. The synthesis and release of hyaluronic acid (HA) may be a mechanism by which PMCs regulate inflammation. Evidence indicates that HA released by PMCs in the peritonitis inflammatory environment can sequester free radicals and initiate repair programs [38]. HA is modified at the injury site to form DAMPs, which can bind to pattern recognition receptors on inflammatory cells to induce inflammatory responses [3,38]. Furthermore, small HA oligomers can promote the expression levels of transforming growth factor-β (TGF-β) and tumor necrosis factor α (TNF-α) leading to the impairment of cellular repair and an increase in inflammation [50]. PMCs can also recruit inflammatory cells. Damaged PMCs can directly attract neutrophils and monocytes to the injury site by upregulating C-X-C motif ligand 1 (CXCL1), monocyte chemoattractant protein 1 (MCP-1), and other chemokines in the early stage of PAs, causing inflammation [42]. The pro-inflammatory effect of PMCs was also confirmed in a study where PMCs were damaged owing to high glucose. Chu et al. reported that PMCs under high glucose conditions activate the MAPK pathway by the autocrine high mobility group box 1 (HMGB1) to stimulate the excretion of MCP-1 and interleukin-8 (IL-8), thereby amplifying the inflammatory response [60]. In conclusion, PMCs may initiate the inflammatory response by releasing chemokines and recruiting other inflammatory cells to amplify the inflammatory response, thereby accelerating the formation of PAs.

PMCs may also be affected by the inflammatory environment and further promote the inflammatory response (Figure 1B) [59]. Terri et al. found that under the action of cytokines, PMCs recruited leukocytes by upregulating surface adhesion molecules such as ICAM-1 and VCAM1 [61]. There is evidence that fibrin can induce PMCs to express IL-1β, IL-6, TNF-α, and vascular endothelial growth factor-A (VEGF-A) to promote peritoneal inflammation and PAs [59]. After surgical trauma, the expression level of IL-22 receptors on the surface of PMCs increases. Upon binding with IL-22 secreted by immune cells, PA formation is promoted. Moreover, the expression of the IL-22 receptor is upregulated after stimulation of PMCs with interferon-γ (IFN-γ) [62]. Under inflammatory conditions, the expression of protein kinase Cα in PMCs increases and mediates the release of inflammatory mediators from PMCs, promoting peritoneal angiogenesis and fibrosis [63]. The effect of inflammation on PMCs has also been explored in long-term peritoneal dialysis and peritoneal dialysis-associated peritonitis. Some studies have found that inflammatory factors and fibrotic mediators such as TGF-β1 and interleukin-1β can reduce the secretion of decorin by PMCs by increasing the activation of the p38 MAPK and AKT/PI3K pathways, resulting in an excessive deposition of fibronectin secreted by PMCs, causing fibrosis [64]. In addition, mesenteric MSCs can transdifferentiate into macrophages under an inflammatory environment, producing pro-inflammatory factors such as TNF-α [65] and IL-6 [66]. The above evidence suggests that under an inflammatory environment, PMCs can promote the inflammatory response and accelerate the formation of PAs by releasing inflammatory mediators, producing chemokines, and upregulating surface receptors and transdifferentiation pathways.

During PA formation, the production of inflammatory mediators regulates the ECM [43]. CXCL1 may be an important pro-angiogenic agent [67]. Catar et al. showed that CXCL1 secreted by PMCs directly promotes human microvascular endothelial tube formation [67], and VEGF accelerates the PA process by participating in angiogenesis [68,69]. CXCL1 can also upregulate the expression of PAI-1 and promote fibrin deposition [3]. CXCL2 and IL-6 produced by PMCs can recruit and activate neutrophils. IL-6 can promote neutrophils to secrete TNF-α, which in turn stimulates neutrophils and macrophages to produce more TNF-α [43]. In addition, IL-6 induces peritoneal inflammation and fibrosis through a STAT3-dependent pathway; the inhibition of IL-6 can alleviate fibrosis [70]. IL-22 promotes PA formation by stimulating PMCs to release more PAI-1 and by inhibiting the production of t-PA to allow the excessive deposition of fibrin [62].

Although both surgery and peritoneal dialysis can damage PMCs and cause inflammation, there are distinct differences between the two. Intestinal bacteria can move into the peritoneal cavity after surgery, exacerbating the inflammatory response at the surgical site and stimulating the formation of PAs [71]. Inflammation is persistent and acute and may be amplified continuously throughout the progression of surgically-induced PAs. The persistence of the inflammation may be related to the surgical injury itself, as studies have shown that surgical injury causes excessive aggregation and dysfunction of macrophages in the peritoneal cavity [40]. On the other hand, the deposition of fibrinolysis is impeded, attracting inflammatory cells to aggregate, amplifying the inflammatory response, and accelerating the formation of PAs.

In conclusion, PMCs can secrete stimulatory and inhibitory molecules of the plasminogen activation system, inflammatory cytokines, and ECM proteins to participate in the inflammatory response after injury [72]. Moreover, they are involved in a positive feedback loop, whereby they are regulated by the inflammatory environment and themselves further amplify the inflammatory response.

### 4.4. PMCs Develop MMT and Promote Peritoneal Fibrosis

PMCs can participate in fibrosis by secreting ECM components and promoting PA formation through the MMT process (Figure 1C). Activated PMCs are able to produce large amounts of fibronectin and collagen and promote tissue remodeling by re-expressing contractile proteins. They can also produce matrix metalloproteinases, such as MMP-2, MMP-9, and MMP-14, and matrix metalloproteinase inhibitors, such as matrix metalloproteinase inhibitor 1 and PAI-1, to affect fibrosis [73,74,75]. MMT is an important participant in many fibrosis events, such as idiopathic pulmonary fibrosis [76], liver fibrosis [77], and myocardial infarction scarring [78,79]. MMT was first noticed in peritoneal dialysis and is thought to be the basis for peritoneal thickening and fibrosis [37], followed by the discovery of MMT in PAs [80]. The important aspect of MMT in PMCs is their transdifferentiation, where PMCs are transformed from the epithelial cell phenotype to the mesenchymal phenotype [31,81]. This transformation is manifested by the loss of the apical–basolateral polarity of PMCs and E-cadherin expression, as well as overexpression of α-smooth muscle actin (α-SMA) and vimentin. The PMCs eventually transform into fibroblast-like cells with enhanced migratory ability and production of ECM to promote PA formation [30,33,82]. 

Myofibroblasts have a strong ability to synthesize and secrete ECM and contribute to the development of PAs (Figure 1D). The occurrence of MMT in PMCs may be a direct source of myofibroblasts [83,84,85,86]. Sandoval et al. demonstrated for the first time that myofibroblasts in PAs are derived from the MMT of PMCs and emphasized that MMT contributes to the development of pathologic PAs [80]. PMCs are transformed into myofibroblasts and participate in PA formation driven by epithelial growth factor receptors through a genetic lineage tracing system [83]. Cells present at the end of PAs express platelet-derived growth factor receptor alpha (PDGFRα), indicating that these cells have fibroblast properties. Further studies have shown that most of the myofibroblasts expressing PDGFRα are derived from PMCs [17]. Uyama et al. also found that the proliferation of PMC-derived myofibroblasts promotes PA formation [43]. The above findings confirm that PMCs undergo MMT transdifferentiation into myofibroblasts and are involved in promoting fibrosis and PA formation.

The TGF-β superfamily may be indispensable in PMC-induced fibrosis and the PA formation process [61,87,88]. First, there are many sources of TGF-β in the process of fibrosis. In mouse models of cecum cauterization-induced PAs, neutrophils and myofibroblasts are able to produce TGF-β1 [43]. Additionally, inflammatory factors, such as IL-1β, can promote the release of TGF-β [89]. TGF-β1 is a powerful cytokine that can activate the classical Smad signaling and Smad-independent signaling pathways, such as the MAPK pathway and the small GTPase, RhoA, involved in MMT [90]. TGF-β1 receptor inhibitors can effectively attenuate the MMT of PMCs induced by the TGF-β1 signaling pathway [91]. TGF-β1 can also promote fibrosis and PA formation by upregulating PAI-1 and inducing collagen production [3].

In addition, the Wnt/β-catenin signaling pathway is involved in the MMT of PMCs [92,93,94,95]. The Wnt/β-catenin signaling pathway is upregulated in peritoneal dialysate-induced peritoneal fibrosis; the MMT process is blocked by the use of recombinant human Dickkopf-related protein 1, an inhibitor of the Wnt/β-catenin pathway [32]. The PI3K/AKT pathway also plays a role in MMT. Wang et al. found that AKT is overactivated during MMT [96], and the expression levels of p-AKT and α-SMA in PMCs are significantly inhibited after intervention with the PI3K/AKT pathway blocker wortmannin [97]. RhoA/Rho kinase signaling plays a promoting role in advanced glycation end product-induced MMT in PMCs [98]. These pathways cooperate with the TGF-β1 signaling pathway to improve fibrosis [90].

Oxidative stress may also be an integral part of the occurrence of MMT in PMCs [99,100,101,102]. Mitochondrial-generated reactive oxygen species (ROS) may contribute to the early stages of peritoneal injury under high glucose conditions, and astaxanthin may prevent MMT through its antioxidant and anti-inflammatory effects [103]. In addition, it was found that mitochondrial damage of PMCs in peritoneal dialysis patients leads to an increase in mitochondrial reactive oxygen species (mtROS), which in turn promotes MMT [104]. Moreover, TGF-β1 increases mtROS, which triggers an inflammatory response, changes the phenotype of PMCs, and leads to fibrosis [105,106,107,108].

Although both surgery and peritoneal dialysis promote MMT and fibrosis, there are differences in the mechanisms of occurrence. Biomechanical signaling may play a small role in MMT in peritoneal dialysis and play a major role in acute abdominal trauma [73], suggesting that mechanical damage caused by dialysis tubes and peritoneal dialysis may not be as toxic as with dialysis fluid. Further studies have also provided evidence to support this claim, such as acidic substances in peritoneal dialysis fluid and high concentrations of glucose stimulating the activation of peritoneal renin angiotensin, leading to fibrosis in peritoneal dialysis patients and angiotensin receptor blockers preventing the progression of peritoneal fibrosis and PAs [109]. In summary, although the mechanical stimulation of the formation of dialysis tubes and dialysate in peritoneal dialysis is involved in MMT and fibrosis, their contribution may be inferior to the cytotoxicity of the peritoneal dialysate itself. The mechanical stimulation brought by surgery mainly leads to the MMT and fibrosis of PAs.

## 5. Prevention and Treatment Strategies for PAs from the Perspective of PMCs

### 5.1. Protection and Reconstitution of PMCs 

Noncoding RNAs participate in gene transcription and their intervention with related molecules may play an integral role in protecting PMCs. In a model of lipopolysaccharide-induced PA formation, it was found that large intergenic noncoding RNA cyclooxygenase-2 (COX-2) was highly expressed in PA tissues. After inhibiting COX-2, the lipopolysaccharide-induced damage of PMCs was alleviated, and the release of inflammatory factors was reduced. Further studies found that through TLR4/MyD88/NF-κB signaling, COX-2 negatively regulates the injury of PMCs induced by miR-21 [110].

In addition, alanyl glutamine may play a role in protecting PMCs. Glutamine-containing peritoneal dialysate can improve the resistance of PMCs to low-biocompatible peritoneal dialysate and reduce the formation of peritoneal fibrosis [111,112]. Acetylation of HMGB1 in peritoneal dialysis-associated peritonitis may promote PMC apoptosis, and this process is mediated by JNK1 [113]. Therefore, JNK inhibitors may protect PMCs. There is also evidence that general control nonderepressible-2 kinase protects PMCs by reducing the toxicity caused by high glucose to PMCs and inhibits MMT [35].

Mesenchymal stem cells and autologous peritoneal grafts can also promote the reconstitution of PMCs and protect the mesothelial barrier. Studies have found that adipose mesenchymal stem cell-derived extracellular vesicles can promote the proliferation and migration of PMCs, accelerate wound healing, and prevent PAs [58]. In addition, rat bone marrow mesenchymal stem cells were found to reduce inflammation and fibrosis by repairing PMCs [114]. Autologous fat transplantation has an immunomodulatory effect and can be used to treat hypertrophic scars and prevent PA formation [41]. There is strong evidence that fat transplantation prevents the occurrence of PAs by promoting the rapid regeneration of damaged PMCs. Autologous fat transplantation was also found in rat models of cecal wall and peritoneal injury to promote mesothelial healing and reduce PA formation in rats [115]. Autologous peritoneal grafts including PMCs can prevent PA formation by promoting the formation of a healthy peritoneum and mesothelial remodeling of the lesion [116]. The above evidence shows that both mesenchymal stem cells and autologous peritoneal grafts can effectively promote the regeneration of PMCs and play a role in preventing PA formation. Moreover, mesenchymal stem cells and autologous grafts have the advantages of easy access and low immune rejection [41,115,116]. Therefore, mesenchymal stem cells and autologous peritoneal grafts may be the most promising approaches in reducing PA formation by promoting the regeneration of PMCs.

In addition, in vitro-cultured autologous peritoneal PMCs and their ECM scaffolds are feasible options for implantation into an injured peritoneum for repair. Some studies have implanted a designed gelatin-based macroporous flexible cryogel scaffold and scaffold-cultured PMCs into the defective peritoneum. With the complete degradation of the scaffold, the implanted functional PMCs successfully repaired the damaged PMCs [117]. In addition, some groups found that PMCs in peritoneal grafts obtained from sheaths were able to repair the damaged peritoneum. Moreover, further studies found that PMCs need to be located on ECM-containing scaffolds to repair the peritoneum and prevent PA formation [116]. Therefore, the transplantation of PMCs alone cannot prevent the occurrence of PAs, suggesting that suitable stents should be selected when formulating the strategy of transplantation of PMCs for the prevention of PAs.

### 5.2. Preventing MMT in PMCs

As mentioned earlier, MMT is an important player in many fibrosis events. In recent years, MMT has been shown to be involved in the development of tumors, related to tumor progression [118,119,120,121]. Therefore, disease treatment by inhibiting relevant molecules that promote MMT progression has great value.

The use of specific antagonists may have important preventive effects on MMT, such as TGF-β1 antagonists or specific antibodies that block the transdifferentiation of PMCs. The peptide inhibitor P144 of TGF-β1 has been shown to interfere with PA-related MMT and fibrosis in a Smad-dependent manner [80]. The use of anti-mesothelin antibodies in ischemic button mouse models has been shown to effectively reduce PAs by depleting PMCs of adhesion phenotypes [2]. Specific antibodies are highly targeted and have the potential to kill myofibroblasts and prevent early PAs from forming. Therefore, therapies based on specific antibodies to prevent PAs should also be valued by relevant researchers. 

Noncoding RNAs may act in the MMT of PMCs, and their functional regulation can help prevent MMT and alleviate fibrosis and PAs. Using a rat model of peritoneal dialysis, miR-200a was found to target the zinc finger E-box-binding homeobox 1/2 (ZEB1/2) in PMCs to negatively regulate TGF-β1-induced MMT and fibrosis [122]. Another study found that miR-200c could prevent TGFβ1-induced MMT and fibrosis by directly targeting ZEB2 and Notch1 [21]. There are other reports that microRNAs can modulate TGF-β1-induced MMT [123,124,125,126]. The above evidence indicates that some noncoding RNAs may engage in the MMT of PMCs, and intervention at the transcriptional level can effectively prevent MMT and fibrosis.

Some chemicals may also reverse the MMT of PMCs, which is beneficial for preventing peritoneal fibrosis and PA formation. There is evidence that zinc can inhibit the MMT of high glucose-induced PMCs by stimulating the Nrf2 antioxidant pathway and reducing oxidative stress [127]. In addition, hydrogen sulfide can inhibit the high glucose-induced MMT of PMCs through its anti-inflammatory property and by inhibiting TGF-β1-Smad3 signaling pathways [128].

### 5.3. Application of PA-resistant Biomaterials 

Currently, the complications of postoperative adhesions pose a hazard to patients, and more and more research is devoted to the development of biobarrier materials, including films, solutions, and hydrogels, for preventing adhesions [129]. There are currently five materials considered biocompatible and have the advantage of inhibiting PAs. However, using these materials against PAs poses a risk. The first material is found in humans, such as HA and gelatin, and may promote PAs by promoting the proliferation of fibroblasts; the second is starch, cellulose, and other natural molecules that cannot be obtained from the human body, which may cause inflammation and aggravate PAs; the third is synthetic polymers that degrade into small molecules, and acidic substances produced by the metabolism of these polymers may also aggravate PAs; the fourth is polymers, such as polyethylene glycol and polyvinyl alcohol, which have anti-specific protein adsorption properties but are not metabolic and degradable. Finally, inert synthetic polymers with high chemical stability, such as PTFE, may lead to the promotion of the adsorption of collagen on its surface during persistent inflammation [11]. As a result, these barrier materials reduce the incidence of PAs to some extent, but their limitations may reduce their clinical application. The prevention and treatment of PAs through anti-MMT therapy may have advantages.

Firstly, MMT is a pathological mechanism involved in many diseases. Therefore, blocking the MMT of PMCs may not only help prevent PAs but may also treat other concomitant diseases. Second, there are many ways to block MMT, such as using TGF-β1 antagonists to block relevant MMT pathways, designing protocols to intervene in noncoding RNAs, or using drugs to target transdifferentiated PMCs. These protocols do not have surgical area restrictions and toxic side effects. When using a physical barrier to block PAs, the placement of the film may be affected by complex surgical sites [130], or the barrier material may be separated from the surgical site due to weak adhesion force [131].

In summary, the therapeutic strategy based on blocking the MMT of PMCs has great research value. This strategy can fundamentally block the progress of PAs; however, some myofibroblasts do not originate from the MMT of PMCs [132], so the promotion of this strategy may need further exploration. Although biological barrier materials may not be able to be applied in some patients due to immune rejection and other issues, the current development of more suitable liquid or solid barrier materials is ongoing, and many developed materials have also shown great potential, such as albumin-based hydrogels with dynamic and spatial control [133], and bio-targeted photolinkable nanosheets [8]. Considering that barrier materials can carry drugs to the wound site, future studies can combine drugs that block the MMT process of PMCs and barrier materials to maximize the potential of both strategies and avoid the formation of PAs induced by other mechanisms. 

## 6. Summary and Prospects

In conclusion, PMCs may play a central role in causing PAs. PMCs promote PA formation by promoting fibrin deposition and participating in inflammatory responses and fibrosis. These PA mechanisms work synergistically with each other. For example, fibrin deposition promotes inflammation and fibrosis. Nonactivated plasminogen can bind to the receptors on PMCs to promote the ability of PMCs to break through the collagen barrier of the basal layer and promote wound healing and tissue remodeling [72], suggesting that the process of fibrin deposition may affect fibrosis. Fibrin deposition also provides attachment points for myofibroblasts to accumulate, thereby accelerating fibrosis. Another study found that the increase in the amount of fibrin was associated with more inflammatory cells and fibroblast aggregation [134]. In addition, a large number of inflammatory factors and pro-fibrotic factors released during the inflammatory process aggravate the fibrosis process. Owing to the multi-faceted effects of PMCs on PAs, it is important to develop preventive strategies against PMCs. Currently, research is focused on the protection of PMCs, the promotion of PMC reconstruction, and the prevention of the MMT of PMCs. Mesenchymal stem cells and autologous peritoneal grafts may have major advantages in this regard due to their abundant sources and low immune rejection [41,115,116].

To date, there have been many studies on the MMT of PMCs. Numerous studies have demonstrated that PMCs can directly participate in the formation of PAs by producing myofibroblasts through MMT. Myofibroblasts have a strong ability to produce ECM and collagen, which eventually leads to the formation of permanent PAs. However, PAs are formed primarily by tissue-resident progenitor fibroblasts [132]; therefore, the main cells that promote PA formation need to be further studied. In terms of the prevention and treatment of PAs, the current strategies are relatively simple, such as inhibiting inflammation and preventing the MMT of PMCs. However, these strategies may have limited effects. Therefore, it is necessary to further explore strategies and measures to prevent and treat PAs through multiple approaches.

PMCs participate in the occurrence and development of PAs through various mechanisms, all of which involve the release of cytokines and pro-fibrotic mediators. Molecular intervention can most effectively prevent the formation of PAs, without interfering with the normal function of cells [43,62]. This suggests that studies on the molecular mechanisms involved in PA formation should be strengthened in the future to formulate the best prevention and treatment strategies for PAs.

## Data Availability

Not applicable.
